# Isolation, characterization, and pathogenic mechanisms of a novel *Lawsonia intracellularis* train from Guangdong, China: impacts on digestive enzymes and gut microbiota

**DOI:** 10.3389/fcimb.2026.1896940

**Published:** 2026-08-26

**Authors:** Yibin Zhu, Ao Wang, Lianxiang Wang, Nanshan Qi, Haiming Cai, Dehong Yang, Ziying He, Shenquan Liao, Dongfang Zhao, Mingfei Sun

**Affiliations:** 1 Wen’s Group Academy, Wen’s Foodstuffs Group Co., Ltd., Guangdong Provincial Enterprise Key Laboratory of Healthy Animal Husbandry and Environment Control Xinxing, Yunfu, China; 2 State Key Laboratory of Swine and Poultry Breeding Industry, Guangdong Provincial Key Laboratory of Livestock Disease Prevention, Key Laboratory for prevention and control of Avian Influenza and Other Major Poultry Diseases, Ministry of Agriculture and Rural Affairs, Institute of Animal Health, Guangdong Academy of Agricultural Sciences, Guangzhou, China; 3 College of Animal Science and Technology, South China Agricultural University, Guangzhou, China

**Keywords:** infection, isolation, lawsonia intracellularis, pathogenicity, porcine proliferative enteritis

## Abstract

**Introduction:**

*Lawsonia intracellularis* causes Porcine Proliferative Enteritis (PPE), leading to significant economic losses, yet obtaining isolates for vaccine development remains challenging.

**Methods:**

In this study, a novel *L. intracellularis* strain, designated LIGD01, was isolated from the ileum of a pig with acute hemorrhagic enteritis in Guangdong, China. The strain was stably passaged in McCoy cells for 40 generations.

**Results:**

Phylogenetic analysis of the 16S rRNA gene confirmed its identity, and indirect immunofluorescence assay (IIFA) and scanning electron microscopy (SEM) demonstrated characteristic intracellular colonization and curved rod-shaped morphology. LIGD01 induced intermittent fecal shedding starting at 3 days post-infection (dpi), with seroconversion in all challenged pigs by 21 dpi. Necropsy revealed distinct ileal hyperplasia. Histopathology showed villous blunting, epithelial exfoliation, and inflammatory infiltration. Crucially, infection significantly reduced Average Daily Gain (ADG) (252.21 ± 17.07 g/day vs. 300.98 ± 19.39 g/day in controls, *p* < 0.05) and suppressed the activities of key digestive enzymes (trypsin, lipase, amylase) in the pancreas and intestine. Furthermore, *Lawsonia intracellular* infection characterized by a reduction in beneficial genera (*Lactobacillus, Christensenellaceae*) and an increase in opportunistic pathogens (*Streptococcus, Enterococcus*), concomitant with upregulated pro-inflammatory cytokines (TNF-α, IFN-γ, IL-1α).

**Discussion:**

This study not only provides a new candidate strain for vaccine development but also elucidates the physiological and microbial mechanisms underlying growth retardation in PPE.

## 1 Introduction

Porcine proliferative ileitis is an important intestinal infectious disease caused by *Lawsonia intracellularis* infection, which is characterized by adenomatous hyperplasia of immature intestinal cells in ileum and colon crypts (Resende et al., 2020), it mainly affects the absorption and utilization of nutrients in pigs, resulting in low feed conversion rate and seriously affecting the production performance such as feed to meat ratio and daily weight gain (Jacobson et al., 2010; Arnold et al., 2019). There are three main manifestations of porcine ileitis in clinical practice. Acute ileitis generally causes bloody dysentery. Necropsy typically reveals intestinal mucosal bleeding and hyperplasia, and diseased pigs usually die. Chronic ileitis mainly affects pigs aged 6–20 weeks. The sick pigs have symptoms such as watery diarrhea and emaciation. Anatomical examination shows intestinal mucosal hyperplasia, brain-like wrinkles, and undigested feed in the intestinal cavity. Subclinical ileitis has no obvious clinical symptoms, but it will affect the growth rate of pigs and cause huge economic losses (Lawson and Gebhart, 2000; Vannucci et al., 2019).

*Lawsonia intracellularis* is a Gram-negative bacterium belonging to the family Desulfovibrio. The bacteria are curved or S-shaped, with a size of about 0.25 μm × 1.75 μm (McOrist et al., 1995). It was first observed by Rowland AC et al. in 1973. Since Lawson GH et al. successfully cultured it *in vitro* in 1993, *L. intracellularis* has been prevalent in more than 50 countries and regions (Rowland et al., 1973; Lawson et al., 1993). *Lawsonia intracellularis* can survive in feces at 15 °C for up to two weeks through fecal-oral transmission (Collins et al., 2000). The infection of *L. intracellularis* has caused a huge economic burden in the pig industry worldwide, and has been occasionally reported in a variety of other animals including hamsters, horses, deer, ostriches, rabbits, and chickens (Cooper et al., 1997; Watarai et al., 2008; Page et al., 2014; Mirajkar et al., 2017; Ohta et al., 2017; Yeh, 2023). After infection with *L. intracellularis* in pigs, subclinical ileitis will continue to affect the growth performance of pigs within 10 weeks after infection, during which the feed conversion efficiency is reduced by about 50%. Compared with healthy pigs, the average daily gain of pigs affected by ileitis is reduced by about 17%-84% (Collins and Barchia, 2014). According to incomplete statistics, the economic loss caused by ileitis per fattening pig is about 4.65 dollar, and the annual loss of pig farmers in the United States is 56.1 million dollar (Szabó et al., 2023).

Pig farming is one of the major contributors to global meat production. China is the world ‘s largest producer and consumer of pork. However, in recent years, PPE has increased significantly in large-scale pig farms around the world (Arnold et al., 2019, 2021). The infection rate of *L. intracellularis* in China is also high (Arnold et al., 2019, 2021). Although the etiological diagnosis technology of PPE has been relatively mature, the isolation and culture of *L. intracellularis* is still very difficult (Campillo et al., 2021). The culture conditions of *L. intracellularis* are relatively harsh, and its *in vitro* growth is strictly dependent on sensitive cell lines and requires a specific microaerobic environment (Lawson et al., 1993; Koyama et al., 2006). Only a few laboratories around the world have successfully achieved *in vitro* culture (Koyama et al., 2006; Wattanaphansak et al., 2019; Xiao et al., 2022; Xie et al., 2025). To date, only a limited number of *L. intracellularis* isolates have been reported in China, and the availability of indigenous strains for further development remains insufficient. In this study, a strain of *L. intracellularis* named LIGD01 was successfully isolated from the ileum samples of pigs died of acute bloody dysentery in a pig farm in Guangdong Province, China for the first time, and it was stably passaged in McCoy cells to 40 generations and also clarified that it could reduce digestive enzyme activity, destroy intestinal flora and affect production performance after infecting pigs.

## 2 Materials and methods

### 2.1 Sample collection and processing

Ileal samples were collected from a gilt that died of acute bloody dysentery in a large-scale pig farm in Xinxing County, Guangdong Province. The intestinal mucosa was scraped, and 1 mL of sterile normal saline was added for tissue grinding, and then DNA extraction was performed according to the operating instructions of the nucleic acid extraction kit (Guangdong Biaoyun Biological Technology Co., Ltd, Yunfu, China). Conventional PCR was carried out according to the reported specific primers of *L. intracellularis* (**Table 1**) (Jones et al., 1993; Wattanaphansak et al., 2010), to prove that the infected intestine is positive for *L. intracellularis*. The PCR reaction was conducted using rTaq mix (TaKaRa, Japan). The conditions for the PCR were as follows: pre-denaturation at 95°C for 10 min, followed by cycling conditions of 95°C for 30 s, 55 °C for 30 s, and 72°C for 30 s, for a total of 30 cycles. The reaction was concluded with an extension step at 72°C for 10 min. The PCR reaction system consisted of 12.5 μL of Premix rTaq, 1 μL of upstream primers (10 μM), 1 μL of downstream primer (10 μM), 9.5 μL of ddH_2_O, and 1 μL DNA as template, resulting in a total volume of 25 μL. For the qPCR analysis, the TB Green^®^ Premix Ex Taq™ II (Tli RNaseH Plus) (TAKARA, Dalian, China) was employed, and the reactions were carried out using the Biorad CFX Connect thermocycler (Biorad, Irvine, CA). The remaining intestinal samples were stored at -80 °C for subsequent bacterial isolation.

**Table 1:** Target genes detected in the study and their primers sequences.

Gene	Prime ID	Forward primer(5’-3’)	Product size (bp)	Annealing temperature(°C)	Accession number	Source
aspA	LI-aspA-F	TATGGCTGTCAAACACTCCG	319	55	L08049.1	(Jones et al., 1993)
	LI-aspA-R	TGAAGGTATTGGTATTCTCC				
aspA	LI-aspA-QF	GCTGTGGATTGGGAGAAATC	162	60	NC_008011.1	(Wattanaphansak et al., 2010)
	LI-aspA-QR	CAAGTTGACCAGCCTCTGC				
methylase	Li-ubi-F	GCTCATACCGATTGTGTAATGCA	75	60	AM180252	(Nathues et al., 2009)
	Li-ubi-R	GAAAAACAGGCCGTATCCTTGA				
	Li-ubi-probe	TAGCCACATCAAGTGTTCCAGCTGCAAG				
GAPDH	GAPDH-F	CCAAGGAGAACTCAAGATTTGGC	150	60	XM_021091114.1	(Xu et al., 2023)
	GAPDH-R	TGCCGTGGGTGGAATCATAC				
IL-1α	IL-1α-F	AACCTGGATGAGGCAGTGAAA	108	60	NM_214029.1	
	IL-1α-R	AGCACTCACAAACAGTCGGG				
IL-1β	IL-1β-F	ATGCCAACGTGCAGTCTATG	134	60	NM_214055.1	
	IL-1β-R	CATGCAGAACACCACTTCTCT				
IL-13	IL-13-F	TTGCTCTCACCTGCTTTGGT	122	60	XM_005661643.3	
	IL-13-R	GCATAGGGGTGTCTTCTGGT				
TNF-α	TNF-α-F	GGCCCAAGGACTCAGATCAT	106	60	NM_214022.1	
	TNF-α-R	GGCATACCCACTCTGCCATT				
IFN-γ	IFN-γ-F	GTTTTTCTGGCTCTTACTGC	410	60	NM_213948.1	
	IFN-γ-R	CTTCCGCTTTCTTAGGTTAG				
IL-10	IL-10-F	GAAGACGTAATGCCGAAGGC	121	60	NM_214041.1	
	IL-10-R	AGGGCAGAAATTGATGACAGC				

### 2.2 Cell culture

Mouse fibroblasts were derived from the American Center for Type Culture Collection(McCoy B;ATCC CRL 1696). The cells were cultured in 7% fetal bovine serum (FBS;Gibco, USA) and antibiotic-free Dulbecco ‘s modified eagle medium (DMEM; Gibco, USA). The T25 cell culture bottle was cultured in a humid environment of 37 °C and 5% CO_2_. When the cells grew to 80% confluence, trypsin containing 0.25% EDTA (Gibco, USA) was used for digestion and passage.

### 2.3 Isolation and culture of bacteria

The isolation of *L. intracellularis* was carried out according to the method described previously (Lawson et al., 1993; Xiao et al., 2022; Xie et al., 2025). In short, the infected intestinal mucosa was scraped and added with phosphate buffered saline (PBS) containing 1% trypsin (Sigma, USA;0.1 M, pH 7.4) was ground and the tissue homogenate was incubated at 37 °C for 35 minutes. After incubation, the tissue homogenate was mixed with an equal volume of sucrose-potassium glutamate solution (SPG:0.218 M sucrose;0.0038 M KH_2_PO_4_;0.0072 M K_2_HPO_4_ and 0.0049 M potassium glutamate; pH 7.0) and supplemented with 10% fetal bovine serum. The mixed diluted tissue suspension was filtered through 100/200/400 mesh stainless steel filter, and the filtered suspension was successively filtered through 1.2, 0.8, 0.65 μm pore size filter membrane for the second time to collect the filtrate. The DMEM medium containing 1% L-glutamine, 7% fetal bovine serum and 0.5% amphotericin B was used to dilute the filtrate at a volume ratio of 1:10, and then inoculated into McCoy cells with 30% confluence and incubated in a three-gas incubator (Esco Micro Pte. Ltd, Singapore) containing 8% O_2_, 8.8% CO_2_ and 83.2% N_2_ at 37 °C. Three hours after infection, the medium was replaced with fresh DMEM containing 1% L-glutamine, 7% fetal bovine serum, 0.5% amphotericin B, neomycin (50 μg/L), and vancomycin (100 μg/mL) every 2–3 days. After 7 days of incubation, the infected monolayer cells were incubated with 0.1% KCl solution at 37 °C for 15 min. The incubated monolayer cells were resuspended in 10 mL SPG solution and passed through a 10-inch 21-gauge needle for 8 times to release bacteria. The suspension was centrifuged at 200×g for 10 minutes to remove cell debris and nuclei. The supernatant was centrifuged at 8000×g for 15 minutes and the bacterial culture was harvested is used for subsequent subculture.

### 2.4 PCR identification and sequence analysis

After harvesting the cell culture, the genomic DNA was extracted using the bacterial genomic DNA extraction kit (Tsingke Biotechnology Co., Ltd.). The extracted DNA was used as a template, and the bacterial universal primer 27F/1492R (**Table 1**) was used for 16S rRNA gene amplification. The reaction system (25 μL) was Premix rTaq 12.5 μL (TAKARA, Japan), 10 μM upstream and downstream primers 1 μL each, template 1 μL, and nuclease-free water 9.5 μL. The reaction conditions were pre-denaturation at 95 °C for 3 min. Cycle conditions: 95 °C, 30 s; 55 °C, 30 s; 72 °C, 90 s; 30 cycles; finally, extension at 72 °C for 10 min. The PCR product was purified using Hipure Gel Pure DNA Mini Kit (Magen Biotechnology, China), and the purified PCR product was sequenced (Sangon Biotech Co., Ltd.) to obtain the amplified sequence. ClustalX software (https://bio.tools/clustal2) was used to compare the obtained nucleotide sequence with the reference sequence. Then the aligned sequences were imported into MEGA X software(https://www.megasoftware.net/)to evaluate the best fitting model and parameters based on Akaike. The Neighbor-Joining algorithm was used to perform phylogenetic analysis of the sequence using 1000 bootstrap replicates and the best fitting model, and a genetic evolutionary tree was constructed to evaluate the taxonomic status of *L. intracellularis* isolated in this study.

### 2.5 Indirect immunofluorescence assay

The growth of bacteria in McCoy cells was monitored by indirect immunofluorescence assay (IIFA). McCoy cells were seeded in a 12-well cell culture plate pre-placed with a 20 mm sterile cell climber. After overnight culture to a cell confluence of 30%, the intracellular *L. intracellularis* and strain B3903 (Boehringer-Ingelheim, USA) isolated in this study were inoculated into monolayer cells and incubated at 37 °C for 5 days. After incubation, the infected and uninfected monolayer cells were fixed with pre-cooled methanol for 15 minutes, the fixative was discarded, and the cells were washed three times with PBS. At room temperature, the cells were blocked with immunostaining blocking solution (Beyotime, China) for 60 minutes, and the fixed cells were combined with *L. intracellularis* monoclonal antibody (BIO 323; bio-XDiagnostics, Belgique) was incubated at 37 °C for 1 h. After washing with PBS for 3 times, 1:200 diluted Cy3-conjugated Goat Anti-Mouse IgG (ABclonal, China) was added and incubated in dark at 37 °C for 1 h. After washing with PBS for 3 times, 4,6-diamidino-2-phenylindole (DIPA; Beyotime, China) were incubated in dark at room temperature for 10 minutes to stain the nucleus. Finally, the samples were observed by inverted fluorescence microscope (Nikon, Japan) immediately after washing.

### 2.6 Scanning electron microscope

After harvesting the bacterial culture, the samples were washed three times with PBS, pre-fixed with 2.5% glutaraldehyde, and then washed with PBS to remove the excess fixative. Next, the samples were fixed with 1% OSO_4_ for 3 h, and gradient dehydration and critical point drying were performed with different concentrations of ethanol. Then the dried samples were installed on the sample stage for gold coating. Finally, Hitachi S-3400N-II scanning electron microscope (Hitachi, Japan) was used for observation.

### 2.7 Transmission electron microscope

After harvesting the bacterial culture, the samples were washed three times with PBS, pre-fixed with 2.5% glutaraldehyde, and then washed with PBS to remove the excess fixative. Next, the sample was adsorbed on a copper mesh, negatively stained with 1% sodium phosphotungstate, and then observed using the Hitachi HT7700 transmission electron microscope (Hitachi, Japan) after natural drying.

### 2.8 Animal experiment

In this study, 12 35-day-old weaned Large White piglets (average weight 11.28 ± 0.58 kg, weaned at 21 days of age;Purchased from the Wen ‘s Food Co., Ltd.) were selected and randomly divided into 2 groups using a computer-based random order generator at the Laboratory Animal Center of Institute of Animal Health, Guangdong Academy of Agricultural Sciences, namely the *L. intracellularis* challenge group and the blank control group, with 6 pigs in each group. Each pig as an experimental unit. The two groups were placed in the same test conditions isolated barns respectively. All experimental animals were free to drink water and feed. Trial follow-up records and final autopsy evaluations were performed by professionals who did not know the grouping information. Blood and anal swabs of all experimental animals were collected before challenge, and *L. intracellularis* antigens and antibodies were detected by qPCR and serological tests to ensure that they had not been infected with *L. intracellularis*. The pure culture of the 10th generation of *L. intracellularis* was collected and the bacterial titer was determined according to the method improved by Xie et al. (2025). In short, the bacterial culture was diluted gradiently and inoculated into a 96-well plate containing 30% confluent McCoy cells, with 8 replicates per gradient. After inoculation, the cells were incubated at 37 °C for 6 days, and the medium was changed after 3 days of inoculation. After incubation, the number of CPE wells at each dilution was observed and recorded, and TCID_50_ was calculated according to the method of Spearman-Kärber (Lei et al., 2021). Each pig in the LIGD01-inoculated group was orally inoculated with 10 mL of *L. intracellularis*, containing about 1×10^8^ bacteria (Based on the standard curve fitting equation y = 1E + 11e^-0.645x^ (R2 = 0.9936, where y is the DNA copy number and X is the Ct value), the copy number was calculated.), while each pig in the control group was orally inoculated with 10 mL of SPG buffer. The health status of pigs was observed and recorded every day after challenge, and the clinical symptoms and fecal status were scored according to the criteria of Joens et al. (1997). Fecal samples of pigs in each experimental group were collected every 3 days after infection, and the bacterial load in feces was detected by qPCR (Nathues et al., 2009). Blood samples of pigs were collected once a week, and antibody levels were detected using a commercial *L. intracellularis* ELISA kit (Indicon Biotechnology Co., Ltd., China). On the 21st day after infection, pigs were weighed and euthanized(after intravenous injection of 7 mg/Kg Zoletil^®^50 (Bolaiyuan Biotechnology Co., Ltd.) for deep anesthesia, Incision of carotid artery bleeding to death.)and dissected sampled.

At necropsy, ileal tissues were collected for hematoxylin and eosin (H&E) staining. For microbial analysis, approximately 0.3 g of luminal contents from each of the remaining intestinal segments was snap-frozen in liquid nitrogen and then shipped to Suzhou GENEWIZ Biotechnology Co., Ltd. for 16S rRNA gene sequencing of the gut microbiota. Raw reads were processed with Cutadapt (v1.9.1) and Vsearch (v1.9.6). Paired-end reads were merged (minimum overlap 20 bp), and low-quality reads (ambiguous bases, Phred <Q20, or length <200 bp) were discarded. Chimeras were removed by reference-based alignment. Clean reads were clustered into OTUs at 97% similarity using Vsearch, and representative sequences were annotated against Silva 138 via the RDP classifier (confidence≥0.7). Alpha (ACE, Chao1, Shannon, Simpson, Good’s coverage) and beta (Bray-Curtis, UniFrac-based PCA, PCoA, NMDS, and UPGMA) diversity were computed in QIIME (v1.9.1) and R.

Digestive enzyme activity and mRNA expression levels were determined according to the method of Tingting Xu et al. (2023). In short, about 0.2 g of duodenal contents and pancreas were ground after adding 1 mL of sterile normal saline. After centrifugation of 4000 g of tissue homogenate for 5 min, the supernatant was used to determine the activities of trypsin, lipase and amylase. The activities of lactase, maltase and sucrase in the supernatant of jejunum mucosa were measured. The kit was purchased from Nanjing Jiancheng Bioengineering Institute (Jiangsu, China) and operated according to the instructions. According to the instructions of SevenFast Total RNA Extraction kit (Seven Biotech, China), RNA was extracted from ileum tissue samples. The extracted RNA was reverse transcribed into cDNA using an ABScript III RT Master Mix kit (ABclonal, Woburn, MA, USA). Quantitative PCR (qPCR) was performed to analyze the expression levels of target genes. Primer sequences are shown in **Table 1**. The relative gene expression levels were calculated using the 2^^-ΔΔCT^ method, employing GAPDH as the reference genes. For qPCR analysis, the TB Green Premix Ex Taq II qPCR kit (Takara Bio Inc., Kusatsu, Japan) was employed, and fluorescence quantification was conducted using ABI7500 FAST fluorescence quantitative PCR instrument (Thermo Fisher, USA).

### 2.9 Ethical approval

All the animal procedures carried out in the present study were in accordance with the guidelines of the Animal Ethics Committee (No. YC-PT2024059) of Institute of Animal Health, Guangdong Academy of Agricultural Sciences.

### 2.10 Statistical analysis

Normality of data was assessed using the Shapiro-Wilk test, and homogeneity of variances was evaluated with Levene’s test (all P-values>0.05, confirming both assumptions). One-way ANOVA was used to compare multiple groups, followed by Tukey’s HSD *post hoc* test. Statistical significance was set at P<0.05. All analyses were performed using IBM SPSS Statistics 23.0 (IBM Corp., Armonk, NY, USA). Graphs were generated using GraphPad Prism 8.0 (GraphPad Software, San Diego, CA, USA), and data are presented as means with standard errors of the mean (SEM) as error bars.

## 3 Results

### 3.1 Isolation and identification of *Lawsonia intracellularis* LIGD01 strain

**Figure 1:**
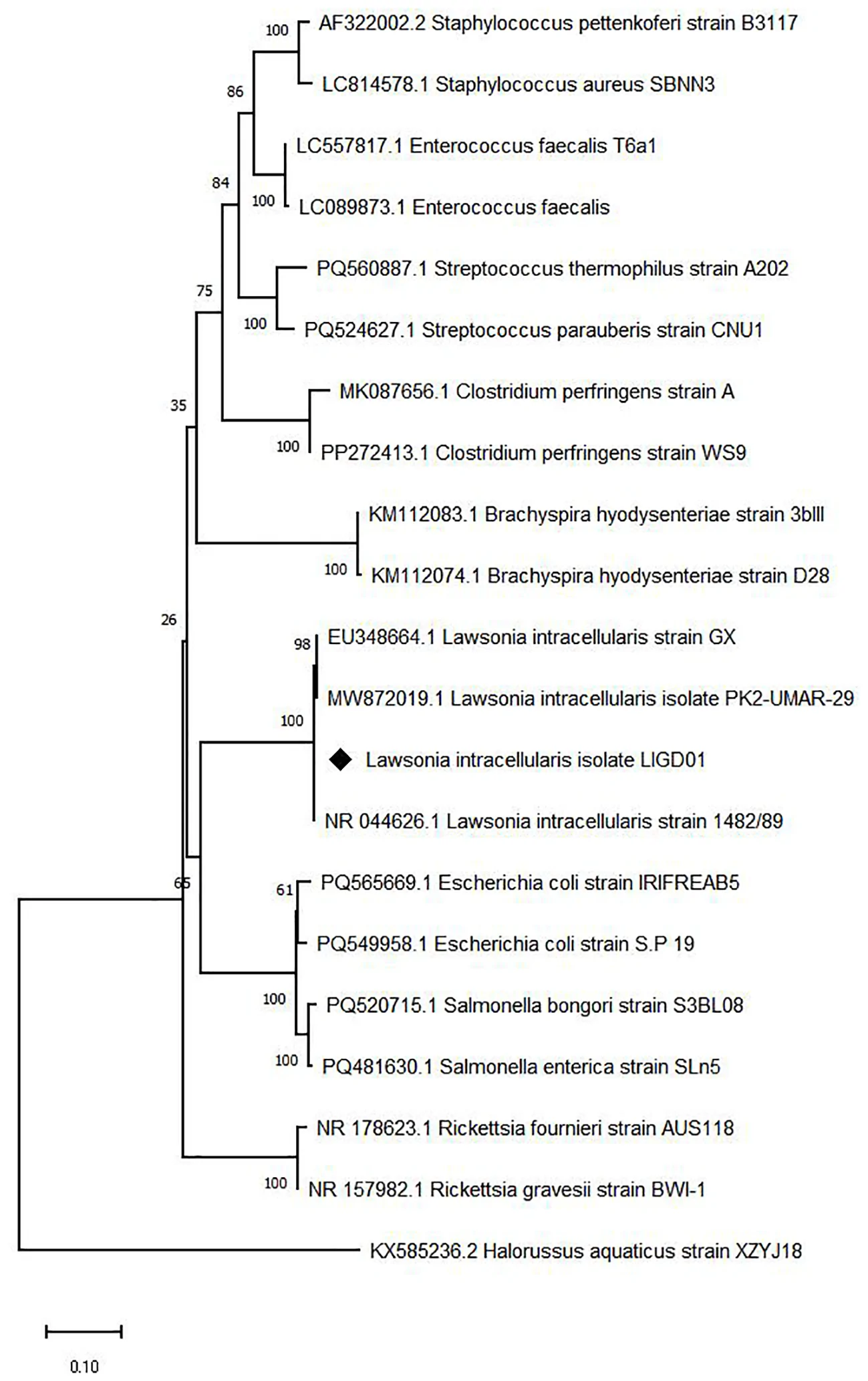
Phylogenetic tree was constructed based on the 16S rRNA gene sequence of *Lawsonia intracellularis*. Some microorganisms in the intestinal tract of pigs were included *Lawsonia intracellularis* (GenBank accession: EU348664.1、MW872019.1、NR_044626.1); *Clostridium perfringens* (GenBank accession: MK087656.1、PP272413.1); *Escherichia coli* (GenBank accession: PQ565669.1、PQ549958.1); *Salmonella* (GenBank accession: PQ520715.1、PQ481630.1); *Rickettsia* (GenBank accession: NR_178623.1、NR_157982.1); *Streptococcus* (GenBank accession: PQ560887.1、PQ524627.1); *Brachyspira hyodysenteriae* (GenBank accession: KM112083.1、KM112074.1); *Staphylococcus* (GenBank accession: AF322002.2、LC814578.1); *Enterococcus faecalis* (GenBank accession: LC557817.1、LC089873.1); *Halorussus aquaticus strain*: KX585236.2 was used as the outgroup.

**Figure 2:**
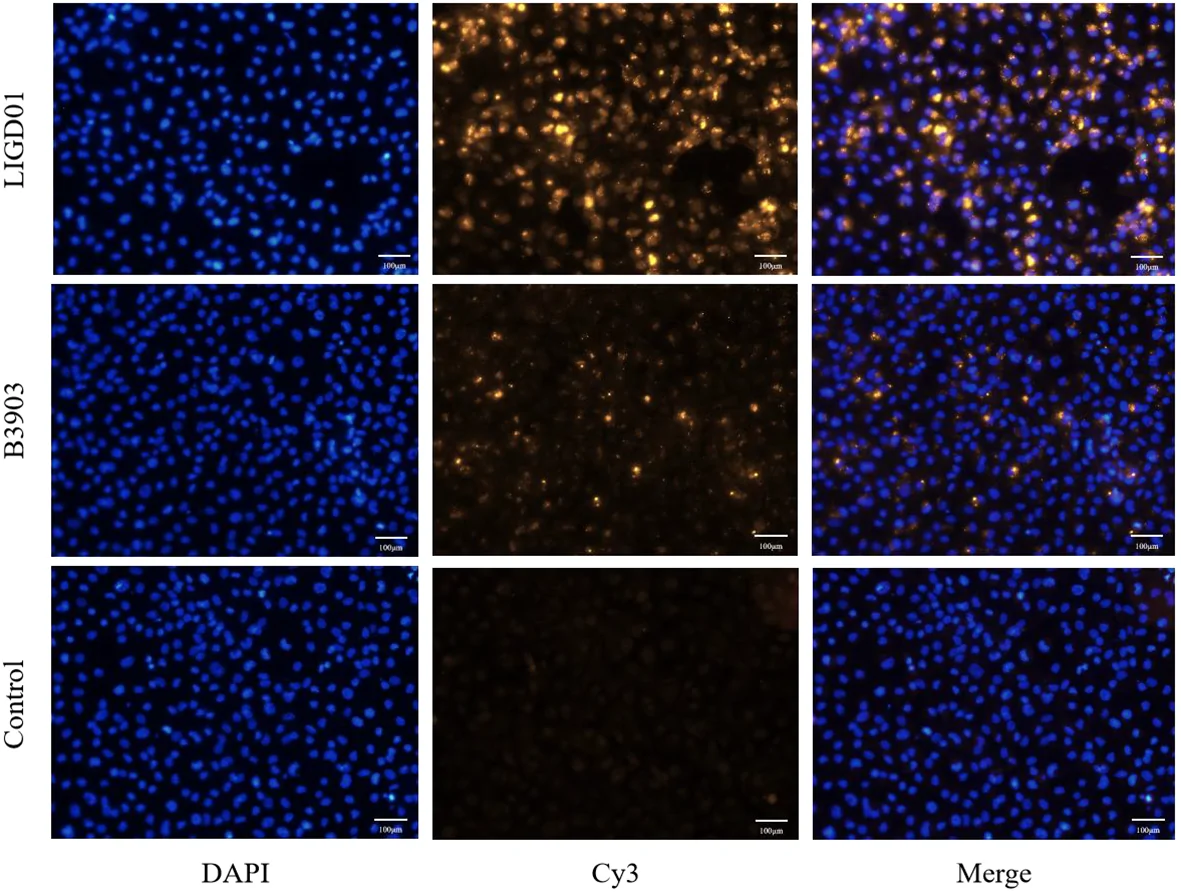
The text result of Indirect immunofluorescence (IIFA).

**Figure 3:**
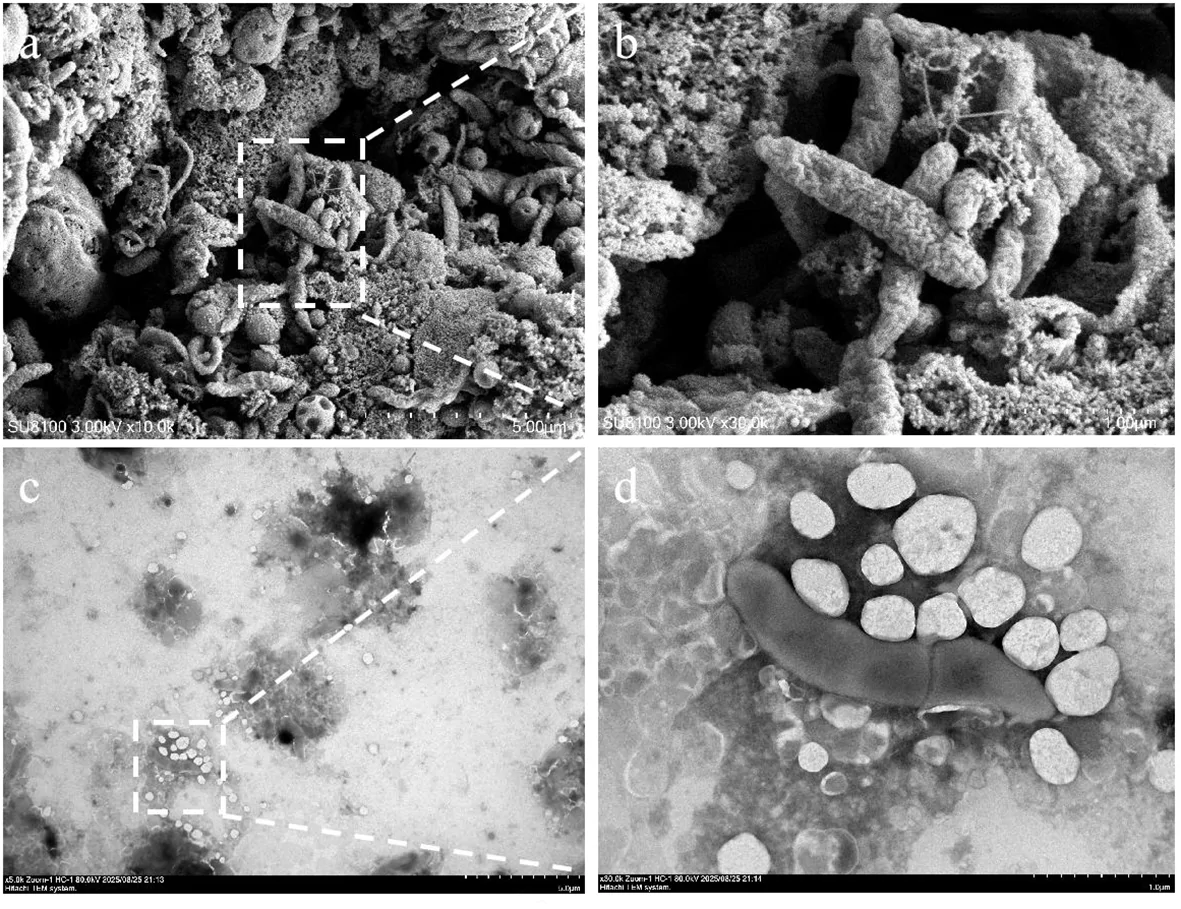
Scanning electron microscopy and Transmission electron microscope was used to observe the morphological characteristics of the pure culture of *Lawsonia intracellularis* LIGD01 (bar=5 μm). **(a, b)** The results of scanning electron microscopy showed that LIGD01 strain. **(c, d)** The results of transmission electron microscopy showed that the LIGD01 strain.

The ileal samples were observed to have obvious intestinal mucosal bleeding symptoms. PCR analysis of the scraped intestinal mucosa yielded a specific band of the expected size. Quantitative PCR revealed a high bacterial load (**Supplementary Figure S1**). After SPG treatment, the bacterial culture was harvested after 7 days of culture, and the quantitative PCR (qPCR) showed a strong positive signal for bacterial proliferation. Three consecutive passages showed that the bacteria showed obvious proliferation. The results showed that a new strain of *L. intracellularis* was successfully isolated from the ileum of a gilt that died of acute bloody dysentery, and was named LIGD01. We successfully cloned the 16S rRNA gene of the *L. intracellularis* isolate. Sequence alignment showed that the 16S rRNA gene homology between the isolate and the *L. intracellularis* PK2-UMAR-29 strain (GenBank accession number MW872019.1) was as high as 99.79%, with only two base mismatches. The results of phylogenetic analysis showed that LIGD01 was clustered within the same clade as *L. intracellularis*, showing the closest genetic relationship (**Figure 1**). The results of indirect immunofluorescence assay showed that the new strain of *L. intracellularis* LIGD01 was localized around the nucleus, but some cells were not infected, which was consistent with the observations for strain B3903. The control group without inoculation of bacteria showed no fluorescence reaction signal (**Figure 2**). SEM revealed that the *L. intracellularis* LIGD01 bacteria were embedded within cellular debris, exhibiting a characteristic curved, rod-shaped morphology without visible flagella (**Figure 3**). The new strain of *L. intracellularis* LIGD01 (CCTCC NO: V202594) was deposited at the China Center for Type Culture Collection.

### 3.2 Animal challenge experiment of *Lawsonia intracellularis* LIGD01 strain

**Figure 4:**
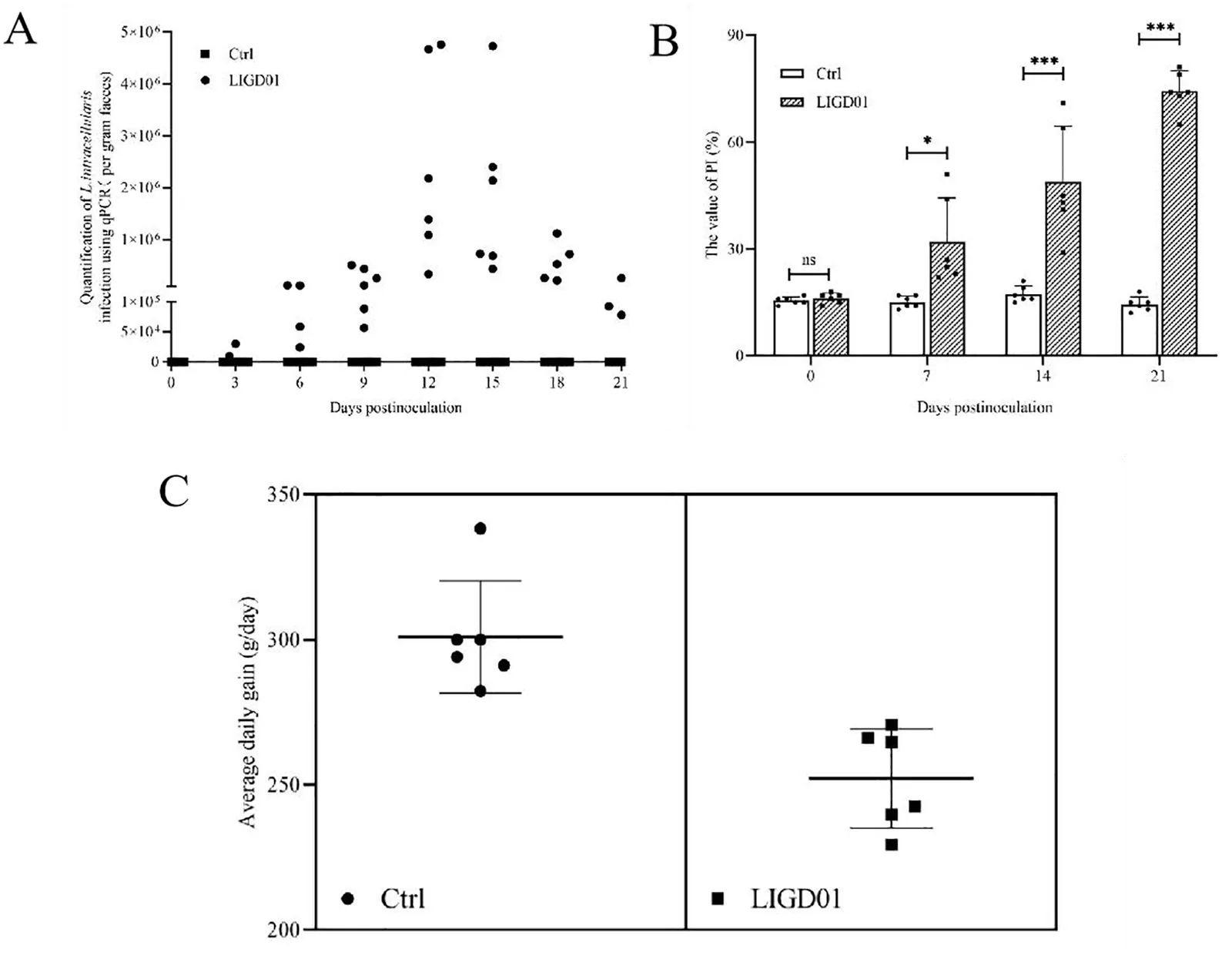
Intracellular *Lawsonia intracellularis* LIGD01 strain animal regression experiment results. **(A)** qPCR was used to detect the content of bacteria in pig manure samples at different times after infection with *Lawsonia intracellularis*; **(B)** The level of specific IgG antibody in pig serum after infection with *Lawsonia intracellularis*; **(C)** The average daily gain of pigs in the infected group and the uninfected group during the test period. (*p < 0.05, ***p < 0.001).

The anal swabs and serum samples of pigs were tested before the beginning of the challenge, and the results showed that the antigen and antibody of *L. intracellularis* were negative, which proved that the pigs had never been infected with *L. intracellularis*. On the 10th day after challenge with the new strain of *L. intracellularis* LIGD01, four pigs developed soft stools and watery diarrhea after infection. After that, intermittent diarrhea was observed until the end of the experiment, but no bloody stools or death occurred throughout the process. The excretion of *L. intracellularis* was detected for the first time on the 3rd day after challenge, and bacterial excretion lasted for about 2–3 weeks. The peak of bacterial excretion appeared 12 days after challenge with the new strain of *L. intracellularis* LIGD01 challenge, with about 1.71×10^6^ bacteria per gram of feces (**Figure 4A**). On the 7th day after challenge, two pigs tested were tested positive for antibodies. At the end of the experiment on the 21st day after challenge, all pigs in the LIGD01-inoculated group turned positive, and during the whole experiment, the antibody test results of the control group showed that PI<30%, showing negative throughout the experiment. throughout the experiment (**Figure 4B**). The average daily gain (ADG) of pigs infected with the new strain of *L. intracellularis* LIGD01 was 252.21 ± 17.07 g/day, which was significantly lower than that of the control group 300.98 ± 19.39 g/day (**Figure 4C**).

**Figure 5:**
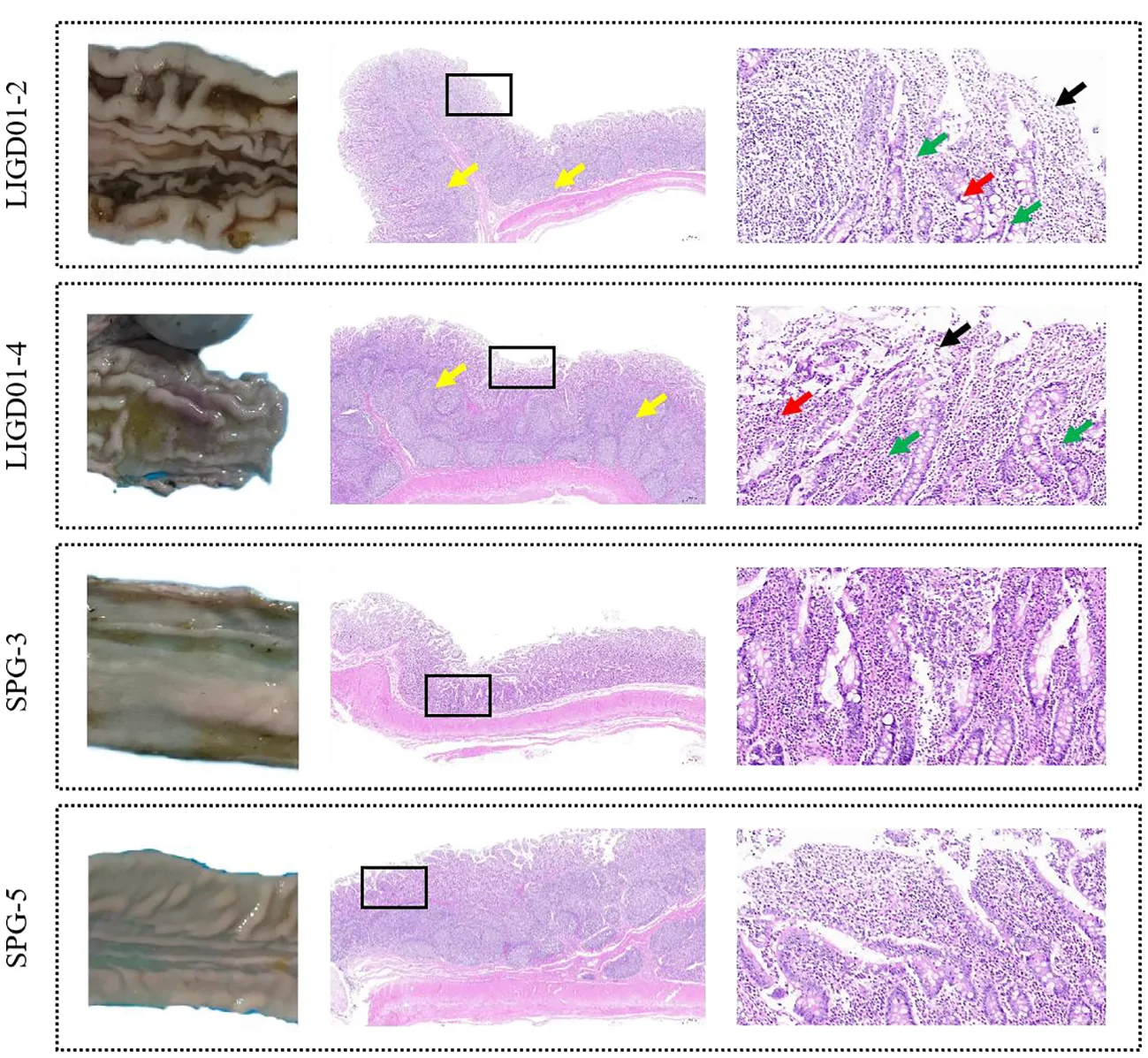
Ileum lesion and histological examination of pigs in the infected group and the uninfected group. LIGD01–2 and LIGD01–4 were infected with *Lawsonia intracellularis*, and SPG-3 and SPG-5 were uninfected. Black arrows indicate the absence of mucosal epithelial cells; yellow arrows indicate lymphoid nodules; the red arrow indicates that the intestinal gland epithelial cells fall off; the green arrow indicates inflammatory cells.

**Figure 6:**
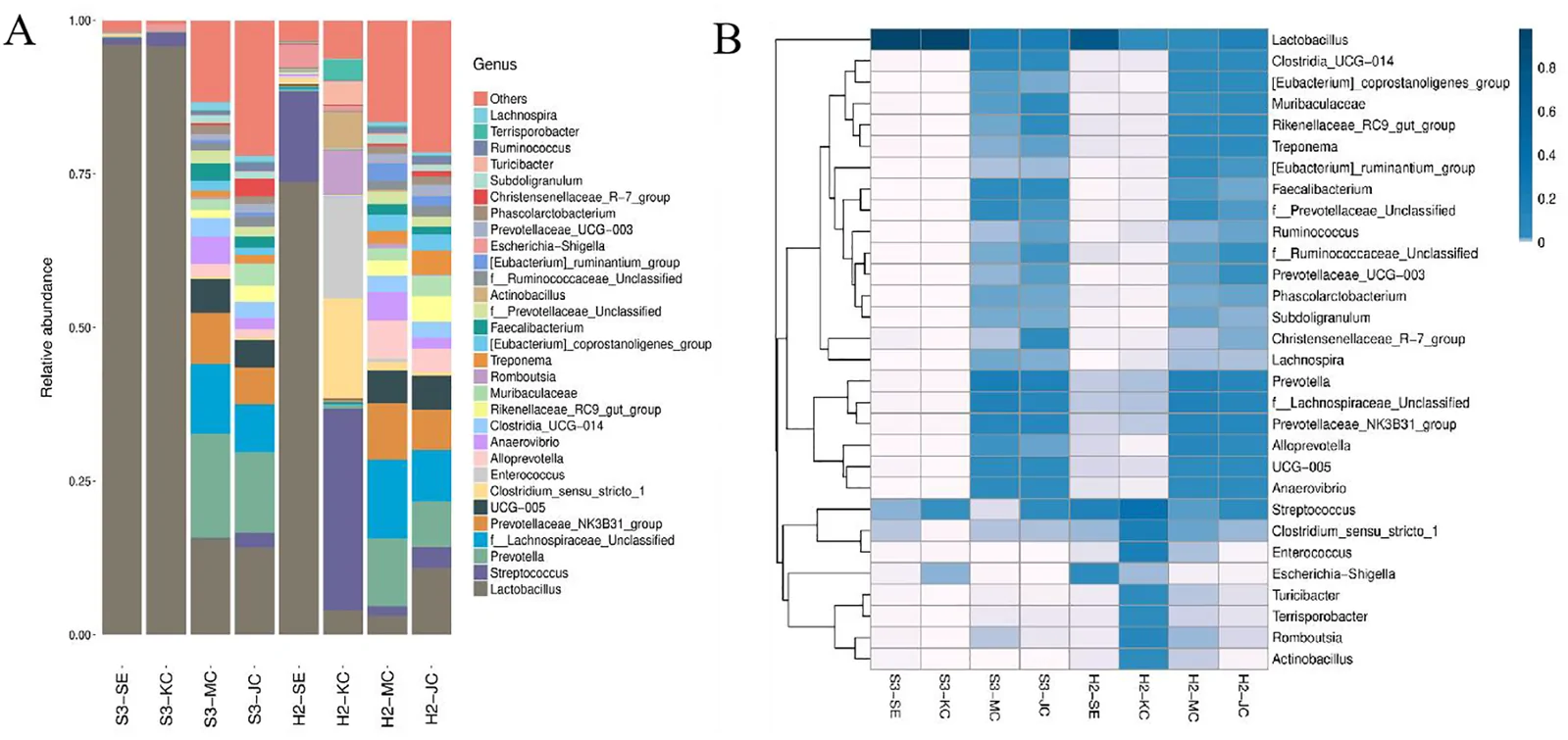
Changes in intestinal flora after infection with *Lawsonia intracellularis* LIGD01 strain. **(A)** Heat map of species distribution in different intestinal segments after infection with *Lawsonia intracellularis* LIGD01 strain. **(B)** Bacterial abundance map of different intestinal segments after infection with *Lawsonia intracellularis* LIGD01 strain. S3-SE, S3-KC, S3-MC and S3-JC were the duodenum, jejunum, cecum and colon of uninfected pigs, respectively. H2-SE, H2-KC, H2-MC and H2-JC were the duodenum, jejunum, cecum and colon of infected pigs, respectively.

The results of autopsy and pathological changes were descriptive, it was found that the ileum of pigs in the infected group showed significantly proliferated, and there was a small amount of undigested feed in the intestinal cavity. Histological examination revealed disrupted villous architecture, exfoliation of glandular epithelial cells, and significant infiltration of inflammatory cells (lymphocytes and plasma cells) into the lamina propria (**Figure 5**). The abundance of probiotics such as *Lactobacillus* and *Christensen ‘s bacteria* in the intestinal tract of pigs infected with *L. intracellularis* showed a decreasing trend, while the abundance of *Streptococcus* and *Enterococcus* showed a trend of higher abundance than that of the control group. (**Figure 6**).

**Figure 7:**
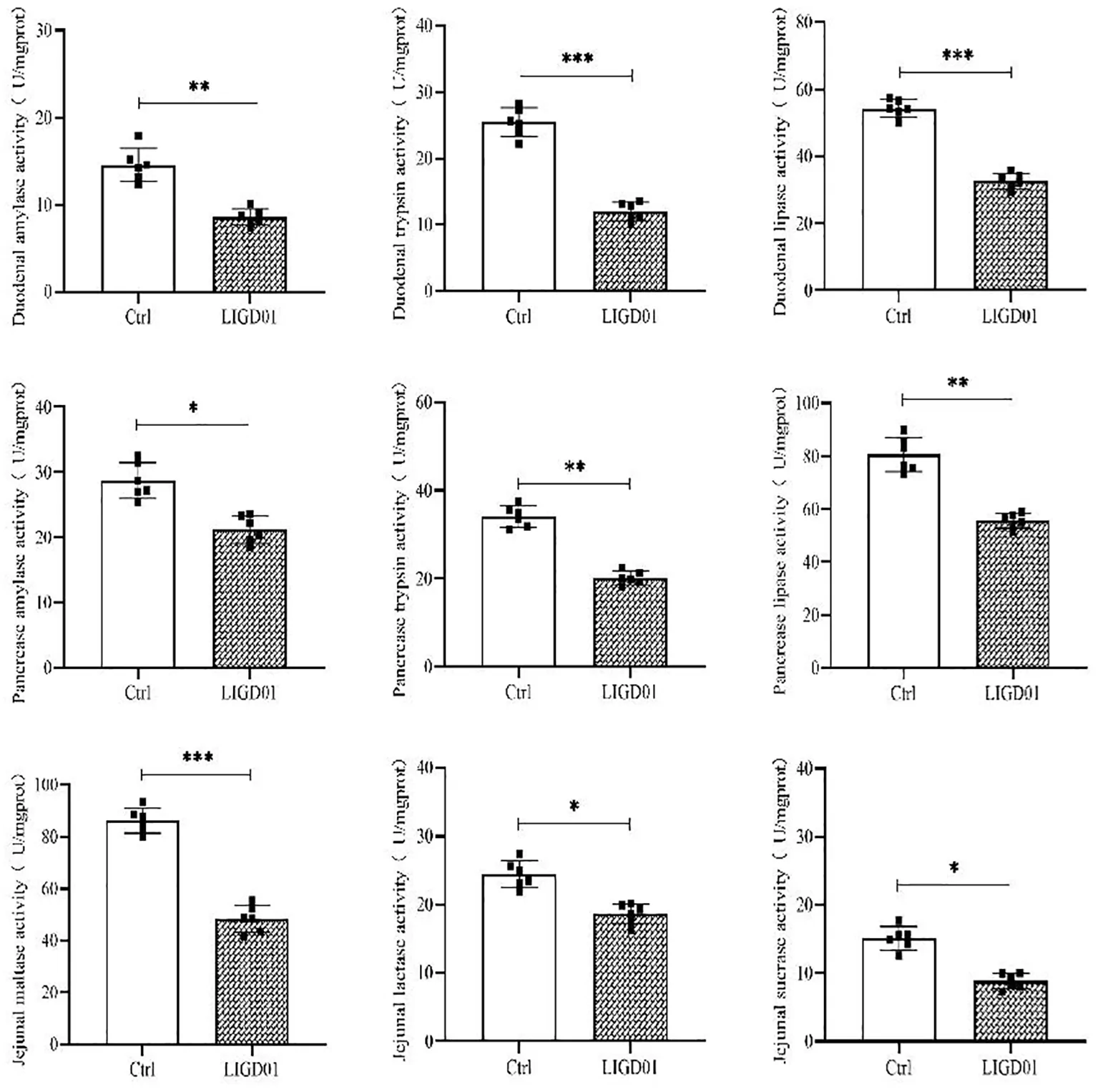
Determination of digestive enzyme activities in pancreas and duodenum of *Lawsonia intracellularis* LIGD01 infected group and uninfected group. (*p < 0.05, **p < 0.01, and ***p < 0.001).

**Figure 8:**
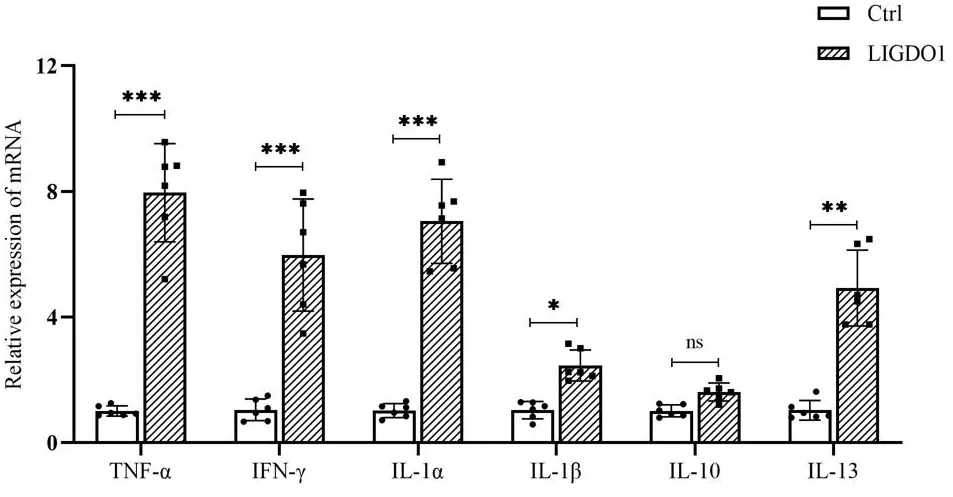
Determination of mRNA expression levels of various typical inflammatory cytokines in ileum tissues. (*p < 0.05, **p < 0.01, and ***p < 0.001).

Notably, LIGD01 infection caused a systemic suppression of digestive function. Activities of pancreatic enzymes (trypsin, lipase, amylase) in the duodenum were significantly lower compared to the control group (*p* < 0.05). Similarly, jejunal mucosal brush border enzymes (lactase, maltase, sucrase) exhibited markedly reduced activity (**Figure 7**). The mRNA detection results of cytokines in ileum samples revealed the degree of inflammatory response. *L. intracellularis* infection increased the expression of inflammatory factors, including the expression of pro-inflammatory factors IL-1α and IL-1β, and the expression of anti-inflammatory factors IL-10 and IL-13. It also significantly increased the expression of TNF-α and IFN-γ (**Figure 8**).

## 4 Discussion

Porcine proliferative enteritis, caused by the obligate intracellular bacterium *L. intracellularis*, continues to impose substantial economic losses on the global swine industry. The pathogen’s fastidious nature, requiring specific environmental and cellular conditions for growth, has been the single greatest impediment to advancing fundamental research and developing efficacious live vaccines. The successful isolation and stable *in vitro* cultivation of a novel strain, designated LIGD01, from a clinical case in Guangdong, China, as reported in this study, therefore represents a significant achievement. This success is underscored by the fact that only a limited number of laboratories worldwide have mastered this challenging technique (Koyama et al., 2006; Wattanaphansak et al., 2019; Xiao et al., 2022; Xie et al., 2025). Our work extends beyond mere isolation; it provides a comprehensive characterization of the new strain of *L. intracellularis* LIGD01, integrating molecular phylogeny, morphological analysis, and a detailed *in vivo* pathogenicity study. This multifaceted approach not only validates the identity and virulence of our isolate but also yields profound insights into the complex pathogenic mechanisms by which *L. intracellularis* disrupts intestinal homeostasis, linking for the first time in a single model the interplay between digestive enzyme suppression, gut microbiota dysbiosis, and a dysregulated host inflammatory response.

The initial isolation of *L. intracellularis* from clinical samples is a formidable task, fraught with technical hurdles. The bacterium’s obligate intracellular lifestyle necessitates the use of susceptible cell lines, with McCoy cells being one of the few documented to support its growth (Lawson et al., 1993). Beyond this, creating and maintaining a specific microaerobic atmosphere (typically 8% O2) is paramount, as the bacterium cannot proliferate under standard aerobic cell culture conditions. Perhaps the most critical step is the pre-processing of intestinal samples to eliminate the immense burden of contaminating microbes without inactivating the target bacterium. Our protocol, adapted from established methods (Lawson et al., 1993; Xiao et al., 2022; Xie et al., 2025). employed a rigorous sequential filtration through filters of decreasing pore size (1.2, 0.8, and 0.65 μm) followed by the use of a selective antibiotic cocktail (neomycin, vancomycin, and amphotericin B). This delicate balance is crucial in regions like China, where widespread antibiotic use in swine production may select for resistant contaminating flora, making decontamination even more challenging. The new strain of *L. intracellularis* LIGD01 has been stably passaged to 40 generations in McCoy cells, which is a powerful indicator of an optimized and robust culture system. This stability is not a given; many initial isolates are lost after a few passages due to inadequate culture conditions or the accumulation of cellular stress. The success with the new strain of *L. intracellularis* LIGD01 provides a valuable, continuously available resource for future studies, including antigen production, vaccine seed strain evaluation, and long-term pathogenesis research, filling a critical gap in the research infrastructure for PPE in China.

Confirming the identity of an intracellular isolate is a critical step, and we employed a polyphasic approach to achieve this. The 16S rRNA gene sequence, a cornerstone of bacterial phylogeny, showed 99.79% identity with the reference strain PK2-UMAR-29, with the phylogenetic tree robustly placing the new strain of *L. intracellularis* LIGD01 within a monophyletic clade containing all other *L. intracellularis* isolates. This high degree of sequence conservation supports the existing paradigm that *L. intracellularis* exhibits limited genetic diversity, likely reflecting its specialized niche and intracellular existence (Vannucci et al., 2012). When placed in a broader geographical context, the new strain of *L. intracellularis* LIGD01 shows a very close relationship to the only other two reported Chinese isolates—one from Hubei province (Vannucci et al., 2012) and another reported by Xiao et al. (2022). This clustering suggests a possible common ancestry or widespread circulation of a highly conserved lineage within Chinese pig populations. However, it is important to note that 16S rRNA gene analysis, while excellent for classification, has limited resolution for discerning fine-scale variations that may influence virulence or antigenicity. Future work involving comparative whole-genome sequencing of the new strain of *L. intracellularis* LIGD01 with other global isolates [e.g., from the USA, Europe, and Brazil (Wattanaphansak et al., 2019)] will be essential to uncover potential single nucleotide polymorphisms (SNPs) or differences in genomic islands that might correlate with phenotypic differences, such as the severity of clinical signs or efficacy of existing vaccines.

Visualization of the bacterium within its host cell environment provides irrefutable confirmation of its intracellular nature and typical morphology. The IIFA results demonstrated a characteristic pattern of the new strain of *L. intracellularis* LIGD01 replication within the cytoplasm of McCoy cells, forming distinct foci around the nucleus. The specificity of this signal, achieved using a commercial monoclonal antibody, confirms that the new strain of *L. intracellularis* LIGD01 expresses the key antigens recognized by the current diagnostic and immunological tools. This is a critical point for validation, as it ensures that research on this isolate is directly relevant to the field situation. The curved rod-shaped morphology observed under SEM is entirely consistent with the established description of the bacterium as being “curved or S-shaped” (McOrist et al., 1995). However, a notable observation was the absence of visible flagella. This structure has been described in some studies, where it is postulated to aid in motility and dissemination from infected epithelial cells (Vannucci et al., 2019). We speculate that our failure to observe flagella can be attributed to several non-mutually exclusive factors: the flagella may be expressed only during specific phases of the intracellular life cycle not captured during our harvest, they might be fragile and sheared off during the vigorous mechanical disruption required to release the bacteria from cells, or the new strain of *L. intracellularis* LIGD01 may genuinely be non-flagellated under the conditions tested. This presents a fascinating avenue for future investigations into the expression of flagellar genes.

The ultimate test of a successful isolation is the ability to reproduce the disease in a susceptible host. The animal challenge experiment with the 10th passage of LIGD01 unequivocally confirmed its pathogenicity. The clinical and shedding patterns observed—onset of bacterial shedding by 3 dpi, peak shedding around 12 dpi coinciding with the appearance of watery diarrhea, and universal seroconversion by 21 dpi—are textbook examples of the infection kinetics described for PPE (Joens et al., 1997; Guedes and Gebhart, 2003). The model effectively replicated a subacute form of the disease, which is of immense economic importance as it often goes unrecognized at the farm level while silently impairing growth performance. The significant reduction in ADG (252.21 g/day vs. 300.98 g/day in controls) quantifies this impact, aligning with field observations and previous experimental data showing reductions of 17-84% (Collins and Barchia, 2014). The gross and histopathological findings were pathognomonic: ileal hyperplasia, blunting and fusion of villi, loss of epithelial integrity, and a marked inflammatory infiltrate. These lesions directly compromise the intestinal absorptive surface area and functional capacity, providing the structural basis for the observed clinical signs and growth suppression (Jensen et al., 2006; Pedersen et al., 2012).

This study’s most significant contribution is its systematic dissection of the multifactorial pathogenic mechanism, revealing a synergistic triad of disruption that explains the full spectrum of disease. We documented a profound and concurrent suppression of digestive enzyme activities throughout the gut. The reduction in brush border enzymes (lactase, maltase, sucrase) in the jejunum confirms previous reports of enterocyte dysfunction (Chwen et al., 2013; Helm et al., 2021). Crucially, we extend this finding by demonstrating a significant drop in the activity of pancreatic enzymes (trypsin, lipase, amylase) in the duodenum. This suggests that the infection’s impact is not confined to the ileum; it may also exert systemic effects, potentially mediated by inflammatory mediators affecting pancreatic exocrine function or the gut-pancreas axis. This is plausibly mediated through interconnected pathways: systemic dissemination of pro-inflammatory cytokines from the infected ileum, which directly impair acinar cell function; disruption of intestinal barrier integrity, with consequent translocation of bacterial products that amplify systemic inflammation and secondarily damage pancreatic tissue; and perturbation of the gut-pancreas axis via dysregulated enteroendocrine hormone secretion and altered autonomic neural inputs, which disrupt the neurohormonal control of enzyme synthesis and release. Collectively, these pathways offer a plausible mechanistic link between local ileal infection, reduced duodenal enzyme activity, and subsequent impairments in nutrient digestion and weight gain. While our findings suggest a plausible mechanistic link between *L. intracellularis* infection, reduced digestive enzyme activity, and impaired weight gain, this proposed pathogenic cycle is based on correlative observations and requires further mechanistic validation. Our 16S rRNA sequencing data revealed that the new strain of *L. intracellularis* LIGD01 infection triggers a severe dysbiosis. The collapse of beneficial genera like Lactobacillus and Christensenellaceae, known for their roles in maintaining barrier function and metabolic health, and the concurrent bloom of opportunistic pathogens like Streptococcus and Enterococcus, indicate a shift to a pro-inflammatory and less protective microbial community (Marchesi et al., 2016; Wu et al., 2022). This dysbiosis is not a passive consequence but an active driver of pathology. The altered microbiota can further degrade the mucosal barrier, produce toxins, and continuously stimulate the host immune system, thereby creating a vicious cycle that exacerbates and perpetuates the initial damage. The functional significance of the observed microbial changes needs to be further studied in the future. At the molecular level, we observed a potent but seemingly dysregulated inflammatory response. The upregulation of key pro-inflammatory cytokines (IL-1α, IL-1β, TNF-α, IFN-γ) is consistent with a vigorous Th1-type immune response against an intracellular pathogen^[43]^. However, this response is a double-edged sword, responsible for much of the tissue damage. The significant concurrent elevation of the anti-inflammatory cytokine IL-10 is a critical finding. It likely represents a host-driven attempt to control excessive inflammation and mitigate immunopathological damage, a protective feedback mechanism that has been observed in other studies (Xu et al., 2023; Obradovic and Wilson, 2020). The activation of the NF-κB signaling pathway is almost certainly a central orchestrator of this cytokine storm (Yang et al., 2024). The dynamic balance between these pro- and anti-inflammatory forces ultimately determines the clinical outcome.

The power of our model lies in the interconnection of these three pillars. The initial bacterial invasion and inflammatory response damage the epithelium, leading to digestive enzyme loss and creating a permissive environment for dysbiosis. The dysbiotic microbiota, in turn, fuels further inflammation and impedes tissue repair. This self-reinforcing cycle creates a perfect storm that leads to the failure of intestinal function and the profound growth retardation characteristic of PPE.

While this study provides a comprehensive analysis, several limitations should be acknowledged. First, the animal experiment included a relatively small sample size (n=5 per group). Although the sample size was sufficient to detect significant differences between groups, it limits the ability to perform deeper correlative or subgroup analyses, and the findings should be validated in larger, independent cohorts to confirm their generalizability. Second, the gut microbiota data, while revealing structural shifts in community composition, were generated using 16S rRNA gene amplicon sequencing. This approach provides reliable taxonomic classification at the genus level but lacks the resolution to discriminate at the species or strain level, and it does not directly capture functional genes, metabolic pathways, or absolute bacterial abundances. Metagenomic sequencing would be required to comprehensively infer functional changes and to characterize the functional potential of the microbiome in response to infection. Third, the new strain *L. intracellularis* LIGD01 was identified based on 16S rRNA gene sequencing; however, whole-genome sequencing (WGS) was not performed in this study. Consequently, we could not assess the presence of virulence-associated genes, genomic islands, or antimicrobial resistance determinants, which would be valuable for comparing LIGD01 with other isolates. Future WGS of LIGD01 will enable a more comprehensive genomic characterization and help elucidate the genetic basis of its pathogenicity, which would be highly informative for understanding regional strain variation in China. Furthermore, investigating the efficacy of commercial vaccines against LIGD01 challenge would provide crucial practical insights for the local swine industry.

## 5 Conclusion

In conclusion, we have isolated a novel *L. intracellularis* strain, LIGD01, which will serve as a valuable resource for investigating porcine proliferative enteropathy. Our findings integrate digestive dysfunction, microbial dysbiosis, and host inflammation into a unified framework, emphasizing the systemic nature of the disease. Importantly, the availability of a local strain enables critical assessments of vaccine efficacy against regional isolates, while the identified host and microbial factors suggest potential targets for novel interventions. This study thus translates fundamental insights into tangible strategies for controlling an economically significant swine disease.

## Data Availability

The original contributions presented in the study are included in the article/**Supplementary Material**, further inquiries can be directed to the corresponding author/s.
